# Absence of free carriers in silicon nanocrystals grown from phosphorus- and boron-doped silicon-rich oxide and oxynitride

**DOI:** 10.3762/bjnano.9.141

**Published:** 2018-05-18

**Authors:** Daniel Hiller, Julian López-Vidrier, Keita Nomoto, Michael Wahl, Wolfgang Bock, Tomáš Chlouba, František Trojánek, Sebastian Gutsch, Margit Zacharias, Dirk König, Petr Malý, Michael Kopnarski

**Affiliations:** 1 Research School of Engineering, Australian National University (ANU), Canberra, Australia; 2 Laboratory for Nanotechnology, Department of Microsystems Engineering (IMTEK), University of Freiburg, Germany; 3 The University of Sydney, Faculty of Engineering and Information Technologies, School of Aerospace, Mechanical and Mechatronic Engineering, Sydney, Australia; 4 Institute for Surface and Thin Film Analysis GmbH (IFOS), Kaiserslautern, Germany; 5 Department of Chemical Physics and Optics, Charles University, Prague, Czech Republic; 6 Integrated Materials Design Centre (IMDC), University of New South Wales (UNSW), Sydney, Australia

**Keywords:** atom probe tomography, doping, photoluminescence, silicon nanocrystals, transient transmission

## Abstract

Phosphorus- and boron-doped silicon nanocrystals (Si NCs) embedded in silicon oxide matrix can be fabricated by plasma-enhanced chemical vapour deposition (PECVD). Conventionally, SiH_4_ and N_2_O are used as precursor gasses, which inevitably leads to the incorporation of ≈10 atom % nitrogen, rendering the matrix a silicon oxynitride. Alternatively, SiH_4_ and O_2_ can be used, which allows for completely N-free silicon oxide. In this work, we investigate the properties of B- and P-incorporating Si NCs embedded in pure silicon oxide compared to silicon oxynitride by atom probe tomography (APT), low-temperature photoluminescence (PL), transient transmission (TT), and current–voltage (*I*–*V*) measurements. The results clearly show that no free carriers, neither from P- nor from B-doping, exist in the Si NCs, although in some configurations charge carriers can be generated by electric field ionization. The absence of free carriers in Si NCs ≤5 nm in diameter despite the presence of P- or B-atoms has severe implications for future applications of conventional impurity doping of Si in sub-10 nm technology nodes.

## Introduction

The conductivity type and free carrier concentration of a semiconductor can be controlled via doping. Conventional impurity doping requires the incorporation of a suitable foreign atom on a lattice site and its ionization by thermal energy. Therefore, the energetic position of a dopant in the bandgap has to be close to the respective band edges. For Si, typical dopant ionization energies are in the range of ≈50 meV. If the size of the Si crystal approaches the exciton Bohr-radius, strong quantum confinement sets in and the valence- and conduction band ground state energies shift to lower and higher energies, respectively. As a consequence, the dopant ionization energies increase, which decreases exponentially the free carrier density [1]. If a doped Si-nanovolume is embedded in a matrix of lower permittivity (e.g., a dielectric), the dopant charge is not fully screened in the silicon and a Coulomb interaction with its image charge in the dielectric occurs. Irrespective of quantum confinement, this so-called dielectric confinement increased the dopant ionization energy even further [2]. At the nanoscale, the incorporation of an impurity on a lattice site is also subject to an increased formation energy as compared to the bulk, so that despite of thermal activation via, e.g., a high-temperature annealing process a significant fraction of potential dopants will remain on interstitial sites [3]. The decreasing number of Si–Si bonds per Si NC atom is a crucial point for the increase of dopant formation energies [4]. These factors impede efficient impurity doping of Si nanovolumes and complicate applications of Si NCs in devices based on p–n-junctions such as solar cells or light emitting devices [5–6]. Furthermore, semiconductor device fabrication technology nodes target the sub-10 nm scale in the near future, i.e., length scales where the effects described above will appear.

Si NCs of a few nanometres in diameter (i.e., quantum dots) represent a good model system to study doping at the nanoscale. They can be fabricated by various methods [7–9] and doped either during growth [7] or post-growth [10]. A recent review provides a broad overview of all available techniques and approaches [11]. Here, we focus on the Si NC growth via phase separation of PECVD-deposited, P- or B-doped silicon-rich oxide thin films via annealing at high temperatures. Additionally, we focus on comparatively lowly doped samples (on the order of 0.1–1 atom %) to study the classical electronic doping of Si NCs. In contrast, dopant concentrations up to 60% (also referred to as hyperdoping) were shown to induce localized surface plasmon resonances and metal-like free carrier densities [12–15]. The standard PECVD precursor gasses for silicon oxide are SiH_4_ and N_2_O. Since Si-rich oxides have to be grown in O-depletion, some of the N-radicals present in the plasma react with the Si and are subsequently incorporated in the film. The resulting material is inevitably a Si-rich oxynitride (SRON) with in our case ≈10 atom % N [16]. Considering some safety issues, the oxidizing PECVD precursor gas can be replaced by O_2_, which allows for N-free Si-rich oxides (SRO) [17]. In both cases, small amounts of PH_3_ or B_2_H_6_ can be added during deposition to achieve P- or B-doped SRON or SRO, respectively.

In this study, we investigate the structural, optical and electrical properties of P- and B-incorporating Si NCs in both embedding dielectrics. We will show that, despite some minor differences in the four different sample configurations, no free carriers associated to a doping behaviour of P or B are observed.

## Experimental

Superlattices of SiO_2_ and SRO, or respectively, SRON were deposited on Si and quartz glass substrates by PECVD using processes described in [16–17]. Small amounts of 1% PH_3_/Ar, or respectively, 10% B_2_H_6_/SiH_4_ were added to the Si-rich layers (both SRO and SRON) whereas in all cases the SiO_2_ barrier layers remained undoped. All samples were annealed for 1 h in ultra-pure N_2_ in a quartz tube furnace at 1100 °C (SRO) and 1150 °C (SRON). The thicknesses of the Si-rich oxide layers determine the mean size of the Si NCs to be of approximately the size of the initial layer thickness. Samples dedicated to luminescence and electrical measurements were post-annealed in the same furnace in pure H_2_ gas at 450 °C for 1 h to enable the passivation of dangling bond defects [18]. For electrical measurements, MOS capacitors were processed by thermal evaporation of Al-contacts. Molecular Cs^+^ secondary ion mass spectrometry (MCs^+^-SIMS [19]; Cameca IMS-4f) with 3 keV Cs^+^ (for SRO:P/B) and 5.5 keV (for SRON:P/B) Cs^+^ was used to quantify sample composition including the P- or B-concentration by means of a calibrated standard. APT was measured with a LEAP™ 4000X Si (Cameca) with a pulsed UV laser (355 nm, 100 pJ, 250 kHz), a cooled specimen holder (≈40 K) and a chamber pressure of 10^−12^–10^−11^ Torr. The atom detection efficiency is 57%. For data reconstruction IVAS™ software (version 3.6.6) was used. APT specimen (needle-shaped tips attached onto the apex of a Mo support grid) were structured using an Auriga (Zeiss) focused ion beam scanning electron microscope. PL was measured using a LN_2_-cooled CCD camera attached to a single grating monochromator with excitation of a HeCd laser (325 nm line). Low-temperature PL spectra were measured from 5 to 300 K using a single-window continuous-flow liquid-He cryostat. TT-dynamics were measured in a standard pump and probe configuration by a laser system with 100 fs pulse length and 1 kHz repetition rate (Tsunami, Spitfire, Newport). The fundamental 800 nm output was partly used as a probe and partly frequency doubled to 400 nm and used as a pump. The measurements were done at room temperature. *I*–*V* and *I*–*t* was measured under accumulation bias, in dark and at room temperature, with an Agilent B1500A semiconductor device analyser and a Cascade M150 Prober in a shielded dark box.

## Results and Discussion

### Dopant concentration and -incorporation

**Figure 1:**
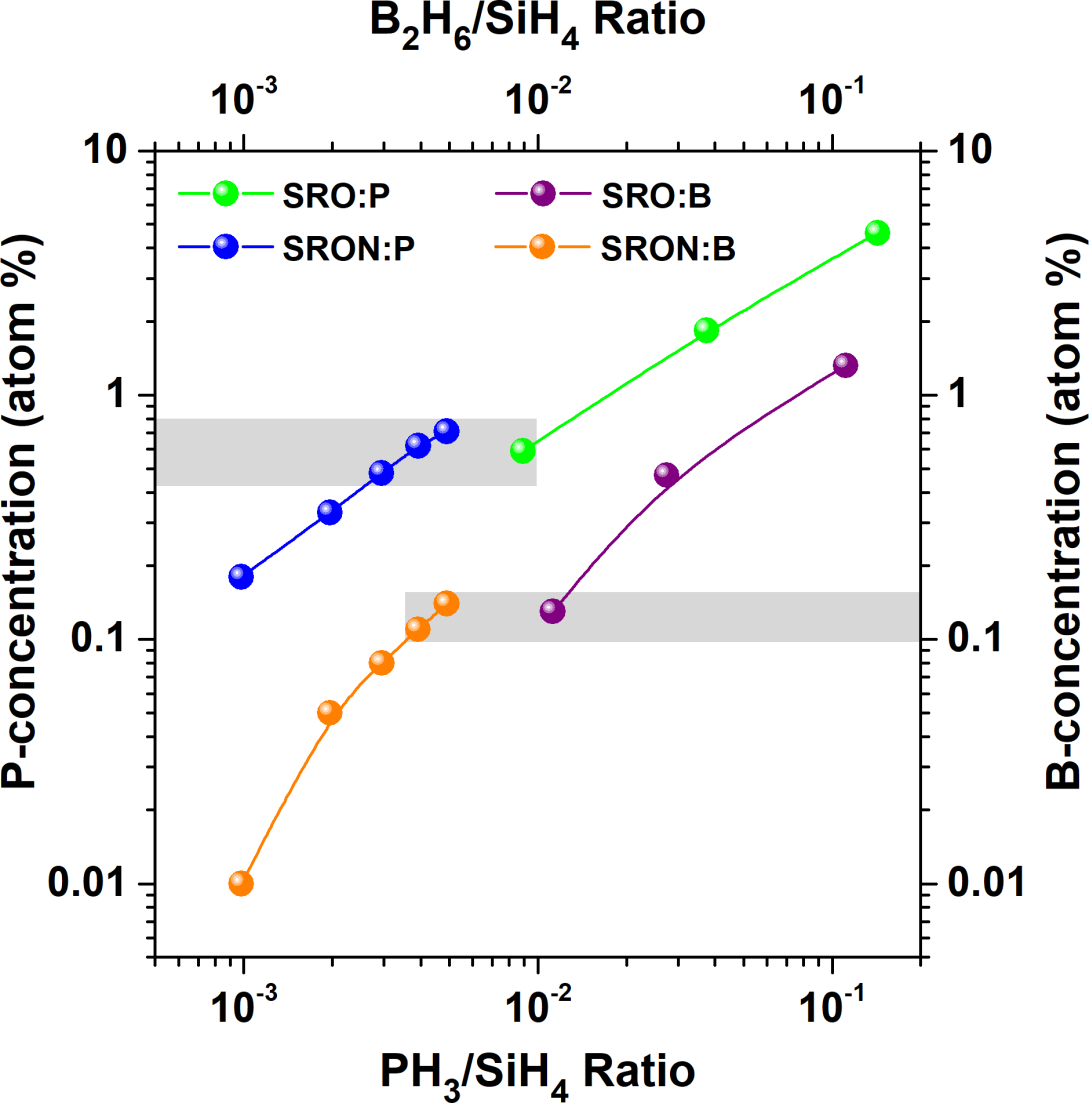
P- and B-concentrations as measured by MCs^+^-SIMS as function of PH_3_ to SiH_4_ gas flow ratio, or respectively, as function of B_2_H_6_ to SiH_4_ gas flow ratio for both SRO and SRON PECVD-recipes. The lines are just a guide to the eye to indicate the nearly linear dependences. The grey boxes indicate the concentration overlap regions for P- and B-doped SRO and SRON materials, respectively.

At first, we determine the P-concentration as function of PH_3_-flux for SRO and SRON via MCs^+^-SIMS measurements. For this task, special samples were fabricated consisting of several 50 nm-thick SRO:P and SRON:P layers with different PH_3_-fluxes, separated by SiO_2_ spacing layers (20 nm and 10 nm thickness, respectively). The SIMS depth profiles for as-deposited SRO:P and SRON:P are shown in Figure S1a and Figure S1b of Supporting Information File 1. It turns out that the P-concentration in SRO:P can be adjusted by the available PH_3_-flux from 0.59–4.61 atom %, while for SRON:P the range is limited to 0.18–0.71 atom %. In Figure S1c and Figure S1d of Supporting Information File 1 the SIMS depth profiles for similarly configured SRO:B and SRON:B layers are shown. Here, the B-concentration is controlled by the B_2_H_6_-flux in the range from 0.13–1.32 atom % for SRO:B and 0.02–0.14 atom % for SRON:B. When plotting the P- and B-concentrations in the Si-rich oxides as function of the flux ratio of PH_3_ and SiH_4_ or B_2_H_6_ and SiH_4_, respectively, a quasi-linear dependence is found; see Figure 1. Generally, the dopant concentrations in SRON are lower than in SRO, which is caused by the very different precursor gas flows used in the SRON [16] and SRO [17] recipes. Nevertheless, for both dopants there is a concentration overlap region (indicated by grey boxes in Figure 1) for P in the range of 0.6 ± 0.1 atom % and for B in the range of 0.13 ± 0.02 atom %. Any direct comparison between doped SRO and SRON samples should hence be made in that overlap region to allow for equal nominal dopant concentrations. While the dopant-precursor flows are similar for each Si-rich oxide type, the average concentration of dopants is a factor of ≈5 lower for B than P, although the same amount of B_2_H_6_ gas contains twice the number of dopant atoms compared to PH_3_. As a consequence, the incorporation efficiency of B in Si-rich oxides is approximately one order of magnitude lower than that of P.

**Figure 2:**
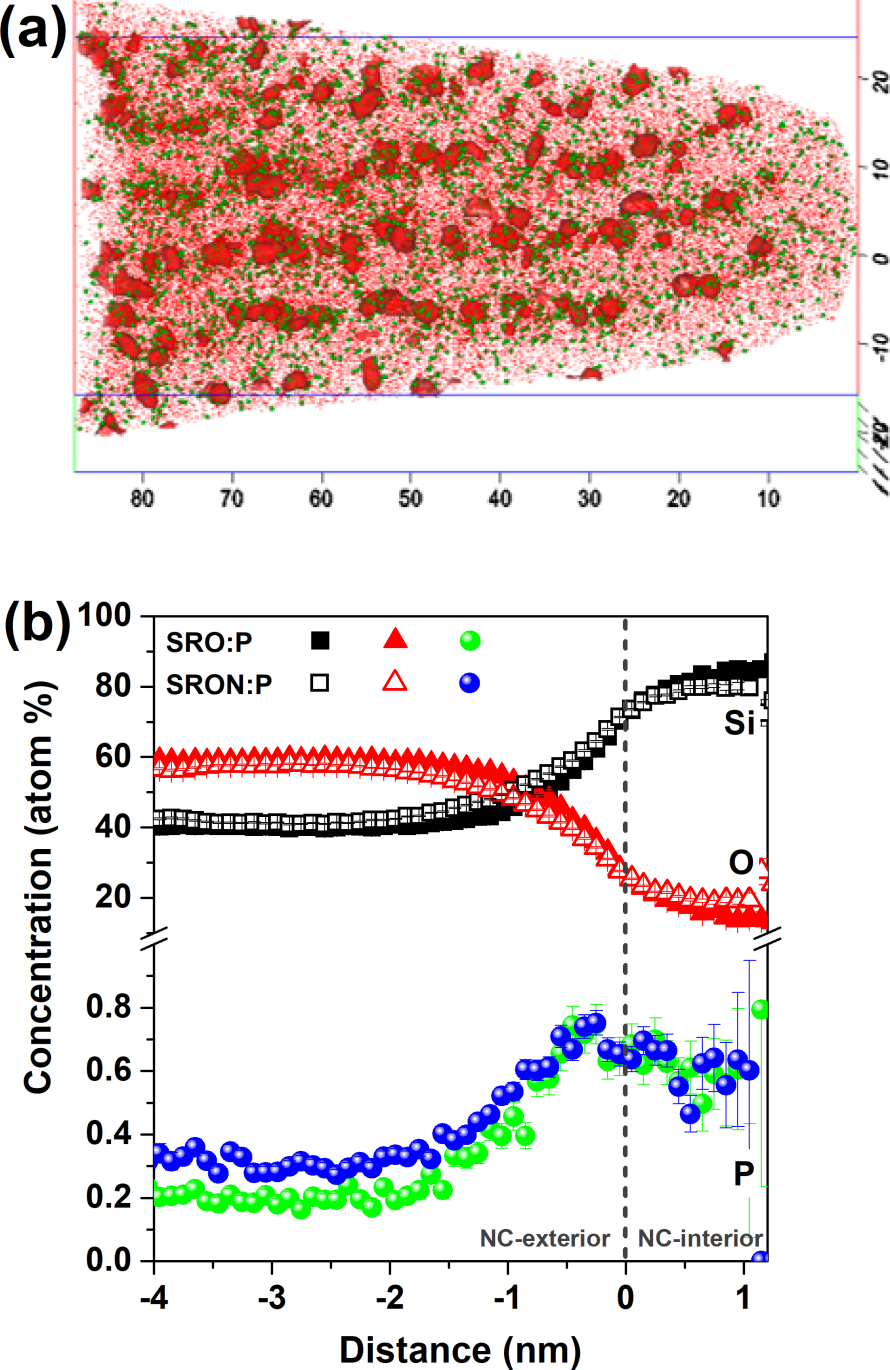
(a) Atom probe tomography reconstruction of P-doped Si nanocrystals (red iso-surfaces with ≥70 atom % Si) in N-free SiO_2_ matrix (P-atoms: green; Si-atoms: red; all axes in nm). (b) Proxigram analysis of Si NCs grown from SRO:P (0.59 atom %) and SRON:P (0.71 atom %). The local P-concentrations are depicted in green for SRO:P and blue for SRON:P. Positive distances refer to the inner NC-volume, negative distances to the surrounding matrix.

Since SIMS cannot reveal the distribution of the dopants in the heterogeneous sample system of Si NCs and SiO_2_ after annealing, atom probe tomography (APT) is used. APT was demonstrated to be a powerful method to reveal structural details of impurity elements in Si NCs [20–21]. In order to determine the incorporation of P-atoms into Si NCs, APT was measured for samples with SRO:P-0.59 atom % (for an image of a typical 3D-reconstruction see Figure 2a) and SRON:P-0.71 atom %. The mass spectra can be found in Figure S2 of Supporting Information File 1. For reference and to exclude critical mass spectra peak overlaps of, e.g., ^31^P^+^, ^30^Si^16^O_2_^2+^, and ^30^Si^1^H^+^ an additional P-free sample was measured and no other signals influencing the ascription to P were found. Furthermore, the signals at 14 Da (Dalton, i.e., the unified atomic mass unit) and 28 Da indicate a very small influence of N on the mass spectra, which is consistent with its rather high ionization energy. Signals of ^14^N^2+^ at 7 Da and ^14^N_3_^+^ at 42 Da in the mass spectra are assigned to N-ion peaks but their contribution is too small to quantify the amount of N. The determination of P-ions in the mass spectra in this study was carried out without further data correction (e.g., for delayed evaporation events, so-called thermal tails). Still, the method to analyse the data of both SRON and SRO samples is the same, thus, P-concentrations are directly comparable to each other. In Figure 2b the proxigram analyses (proximity histograms) [22] of all detected NCs in the respective samples are shown. As selected in previous works, the Si NCs were created by 70 atom % Si iso-concentration surfaces [23]. A voxel size of 0.5 nm and a delocalization value of (*x*, *y*, *z*) = (1 nm, 1 nm, 1.5 nm) were used [24]. The bin size of the proxigram was set at 0.1 nm. Note that these parameters do not change the trend of the composition profiles of both samples. On first sight, no significant differences in the distribution of P-atoms in the NC-interior, at the Si/SiO_2_ interface, and in the SiO_2_ matrix are found. Especially the interior of the Si NCs and the near-interface region of the SiO_2_ have almost identical P-concentrations of ≈0.5–0.7 atom %, while in the N-free SiO_2_ matrix apparently less P is dissolved (≈0.2 atom %) compared to the oxynitride matrix (≈0.3 atom %). However, given the 20% (relative) higher initial P-concentration in SRON:P compared to SRO:P and a measurement uncertainty in the range of 0.1 atom %, this difference might be negligible. The overall P-distribution corresponds very well to previously observed trends for P in Si NCs [23–26]. We note that the ≈20% O-concentration in the NC-interior is an artefact from local magnification effects (LME) [27–28] which is generally observed in this material system [24–26,29–30]. Inevitably, this artefact also influences the exact values of the P-concentration, but since both samples are subject to the same LME the comparison discussed above is not influenced. Besides LME there are also other factors influencing the precision and resolution of APT such as inhomogeneous tip shape evolution during the measurement [31], delayed dissociation of molecules during the flight [32], and associated problems with the detection of neutral fragments [33]. Still, APT provides unique and very useful data inaccessible by any other method.

In Figure S3a of Supporting Information File 1 the NC-size distribution as derived from APT using iso-concentration surfaces of ≥70 atom % Si is plotted as well as the relative frequency of the number of P-atoms per NC. The number of P-atoms incorporated per NC and the P/Si-ratio, both as function of NC-volume, are shown in Figure S3b of Supporting Information File 1. While details of this data set are discussed in Supporting Information File 1, it can be readily concluded that the P-distribution and NC-incorporation is very similar for both SRO:P and SRON:P. Therefore, the presence of N in the oxynitride matrix has apparently no influence on the structural properties of P-doped NC-samples.

The SRO:B material has already been APT-analysed in [30] with the result that B is generally less likely to be incorporated deep in the NC core but more near the inner surface (in agreement with former theoretical [34–35] and experimental [26] evidence). Due to the maximum B-concentration in SRON:B of only 0.14 atom %, a statistically meaningful APT-analysis cannot be achieved. However, the absence of any significant differences between SRON:P and SRO:P suggests that the nitrogen in the oxynitride matrix will not have a notable influence on the B-distribution when SRON:B and SRO:B are compared.

### Photoluminescence and transient transmission

**Figure 3:**
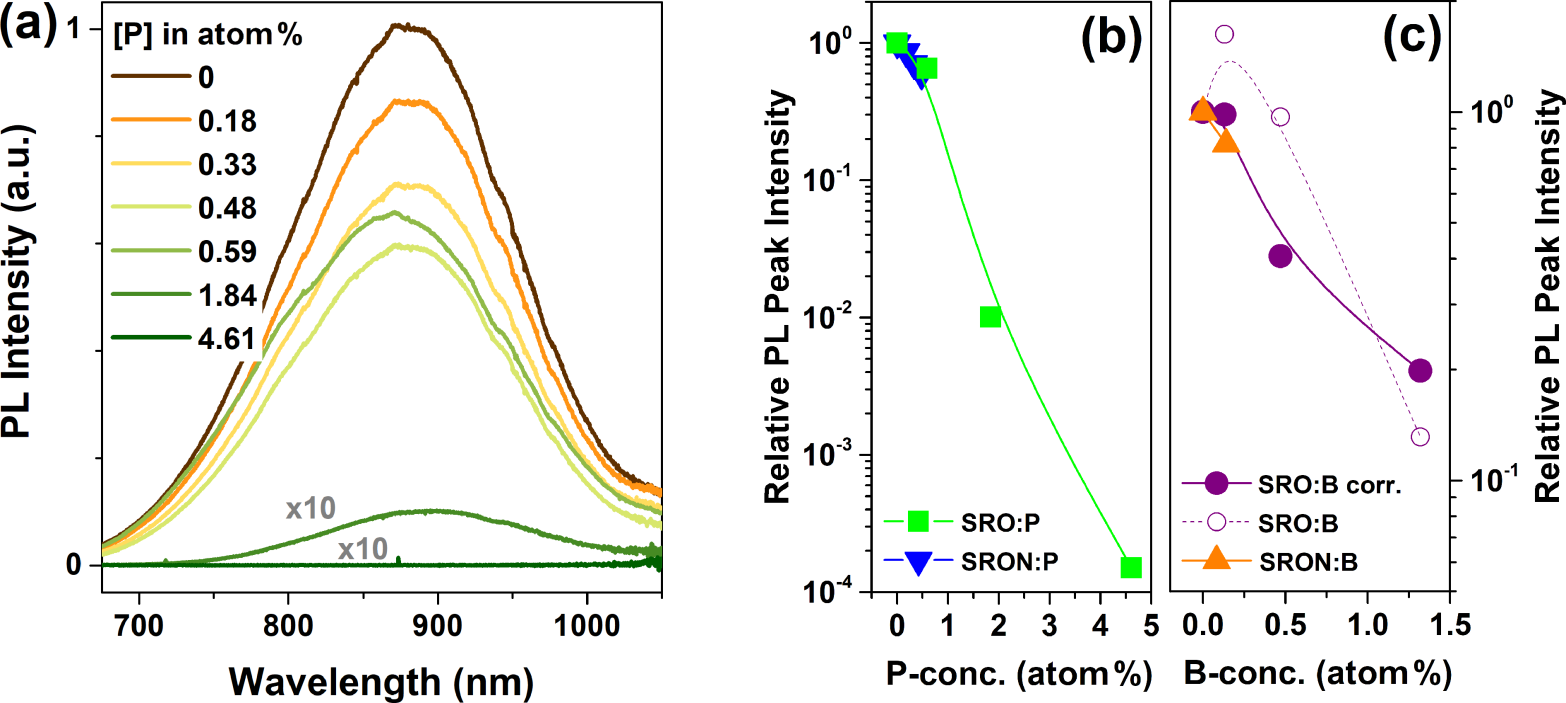
(a) Room temperature photoluminescence spectra of P-incorporating Si NCs in SRON (0–0.48 atom %) and SRO (0.59–4.61 atom %). The spectra of the two highest P-concentrations are multiplied by a factor of 10 for better visibility. (b) and (c) logarithmic PL peak intensity as function of P-concentration, or respectively B-concentration, in SRO and SRON, normalized to the respective dopant-free samples. For SRO:B-samples the PL-intensities are corrected for the excess-Si content (open purple circles show the raw data). Lines are just a guide to the eye. For both dopants it is obvious that significant PL-quenching requires P-/B-concentrations >1 atom %.

Due to quantum confinement effects the ground state energy of Si NCs increases and the k-space overlap of electron and hole wave functions are significantly enhanced (Heisenberg’s uncertainty principle). Therefore, excitons formed in Si NCs are subject to significantly higher radiative recombination probabilities, allowing the luminescence quantum yield to reach ≈30% [36–37], or even ≈60% for organically-capped NCs [38]. In the presence of a third charge carrier (a free electron from an ionized P-donor or a hole from an ionized B-acceptor) radiative recombination is very unlikely, since ultra-fast non-radiative Auger recombination will prevail [7]. On the other hand, the observation of PL quenching alone cannot prove the presence of free carriers since also dopant-induced defects can be involved [29–30,39–41]. In Figure 3a, the dependence of the PL spectra on the P-concentration in SRO:P and SRON:P is demonstrated. Here, all samples are H_2_-passivated and hence only the PL-quenching effect of P-incorporation is visible, not the PL-enhancement often observed for low P-concentrations and associated to dangling bond passivation by P [42]. Up to the level of ≈0.6 atom % P the PL intensity drops by less than 40% without any significant peak shift. According to the APT data shown above and in Supporting Information File 1 only the smallest NCs of each sample remain rather P-free and therefore potentially PL-active, which would implicate a strong PL blueshift, if Auger quenching by P-donors is considered. From Figure 3a and 3b, however, it is obvious that neither a spectral shift nor an efficient PL-quenching by P-incorporation takes place. In contrast, the PL remains very intense up to a P-concentration in SRO beyond >1 atom % P, i.e., vastly exceeding the solubility limit of P in Si. An almost complete suppression of PL occurs only for samples with 4.61 atom % P. Within the concept of PL-quenching by free-carriers induced by P-atoms in the Si NCs, it remains dubious why concentrations of several atom-percent should be required although APT detects in the majority of NCs already one or several P-atoms for samples with 0.6–0.7 atom % P. It appears more consistent with the available data that P-induced defects (e.g., from interstitial P in the Si NCs or SiO*_x_*:P-related states at the surface) cause the PL quenching, as supported by density functional theory (DFT) calculations [29,41]. In that context, it is also likely that for samples with >1 atom % P the P-concentration peak found at the Si/SiO_2_ interface (cf. Figure 2b) reaches a level where a highly enriched P-shell forms on the NC-surface that enables efficient formation of non-radiative defect states.

For boron, strong PL-quenching is also not observed before the B-concentrations exceed >1 atom %, as shown in Figure 3c, and the same argumentation holds true for B-induced defects with states in the fundamental gap of Si NCs, as determined by DFT [30]. The as-measured PL peak intensity of the SRO:B sample set (open purple circles) is unfortunately obscured by variations in the initial excess-Si content (cf. Figure S1c of Supporting Information File 1), which directly influences the NC-density in those samples. Hence, the data set is corrected by the excess-Si content as measured by MCs^+^-SIMS (filled purple circles in Figure 3c; for details see caption of Figure S1 of Supporting Information File 1).

We note that the overall PL-quenching behaviour of Si NCs in doped SRO and SRON is similar. Hence, the presence of nitrogen in the matrix does not have a major impact on the formation of B- or P-induced centres that quench the PL.

**Figure 4:**
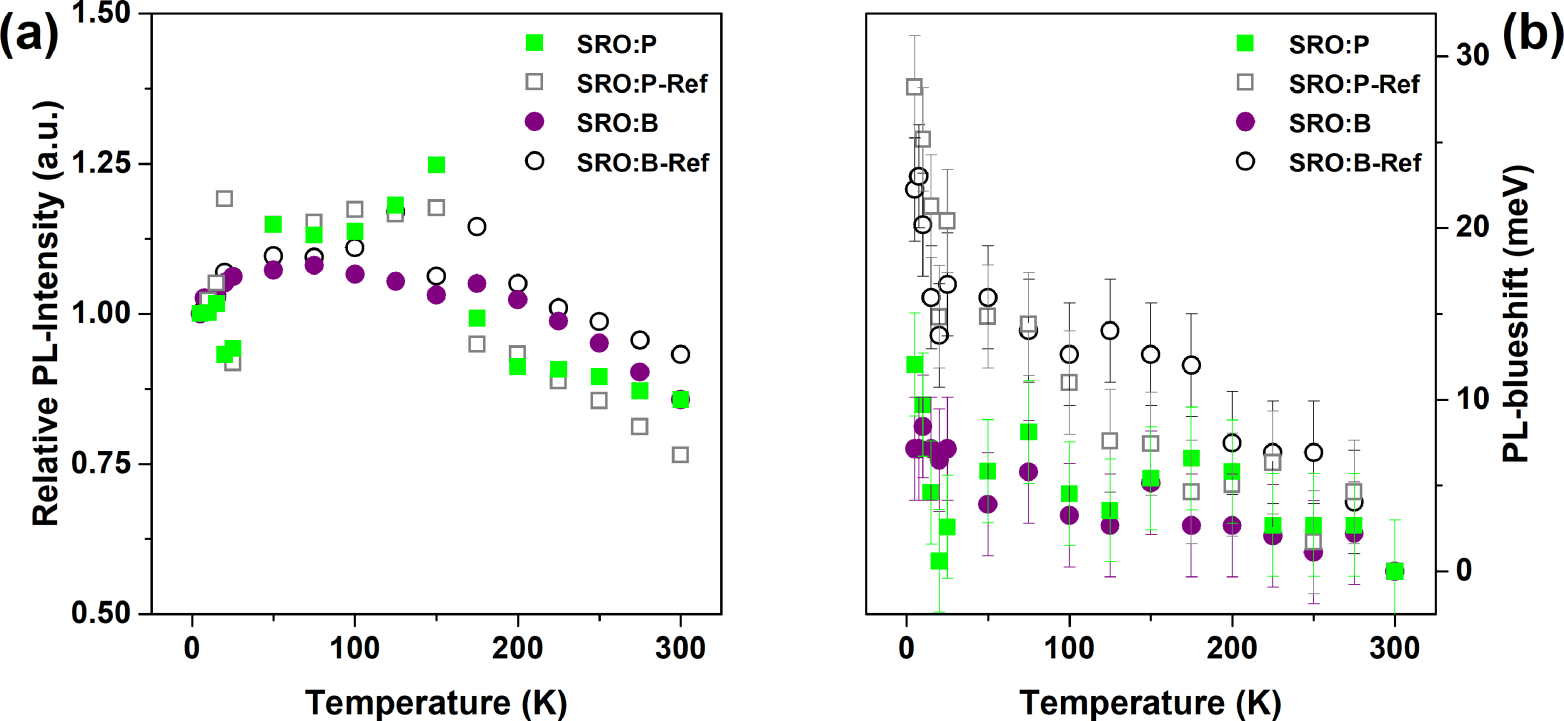
Low-temperature PL data of samples with 5 nm SRO and 0.59 atom % P (SRO:P), 0.47 atom % B (SRO:B) and their respective intrinsic references (SRO:P-Ref, SRO:B-Ref). (a) Relative PL-intensities with respect to the measured value at 5 K of each sample, i.e., the lowest temperature where all potential dopant-induced carriers are completely frozen out. There is hardly any difference visible in the PL-intensity over temperature between doped and undoped samples. (b) PL peak shift with reference to the peak at 300 K, i.e., where ionized donors and acceptors would quench predominantly the largest NCs in each sample. A slight PL-blueshift is observed, not a redshift as expected from NC-doping.

Electronic doping, i.e., the generation of free carriers from dopants on substitutional lattice sites, requires thermal ionization, typically provided by the thermal energy at room temperature. Ignoring all the evidence of a defect-related PL-quenching of Si NCs containing P- or B-atoms, we would anticipate from low-temperature PL measurements of successfully, electronically doped Si NCs: (i) an increase in the PL-intensity as soon as free carriers are frozen out, accompanied by (ii) a spectral redshift due to the circumstance that within the NC-size ensemble the largest NCs are more easily doped than the smaller NCs, and (iii) significant differences in the PL peak behaviour when compared to undoped reference samples. In Figure 4, the T-dependent PL-peak analyses of spectra measured at very low excitation fluxes of ≈0.4 mW/cm^2^ (to prevent over-excitation artefacts [43]) are presented. The spectra themselves can be found in Figure S4 of Supporting Information File 1. Figure 4a shows the relative PL-intensity as function of sample temperature (*T*) with respect to the intensity at 5 K, where all free carriers from potential dopants would be completely frozen out. The intensity trends follow roughly the low-excitation measurements shown in [44]. For approx. *T* > 150 K the intensity drops below unity due to the thermal activation of non-radiative recombination channels [36,44]. The relative PL-intensities of all samples with respect to their 5 K values end up in the same range of values at room temperature. Hence, a freeze-out effect of dopant-induced free carriers that quench the PL is not observed in accordance with dopant-induced defect states deep within the fundamental gap of the NCs.

Figure 4b plots the PL peak shift with reference to 300 K, i.e., where a maximum of dopant-induced free carriers would quench the PL, which would preferentially affect the larger NCs with least confinement energy. Instead of a PL redshift expected for doped NCs with decreasing *T*, we observe a small blueshift related to the thermal contraction of the lattice and reduced electron–phonon interaction, which typically saturates around 100–200 K for lowly excited samples [43]. To add, the blueshift of the reference samples is slightly more pronounced for *T* < 150 K than that of the doped samples. The increase for *T* ≤ 25 K is most likely an artefact from overexcitation [43] despite the very low laser intensity. The reason for using two nominally identical reference samples (both are undoped SRO) in PL is due to the different number of NC-layers in the superlattice (10 for SRO:P and 20 for SRO:B). Any differences between the reference samples might therefore be interpreted as the scattering amplitude between different samples.

**Figure 5:**
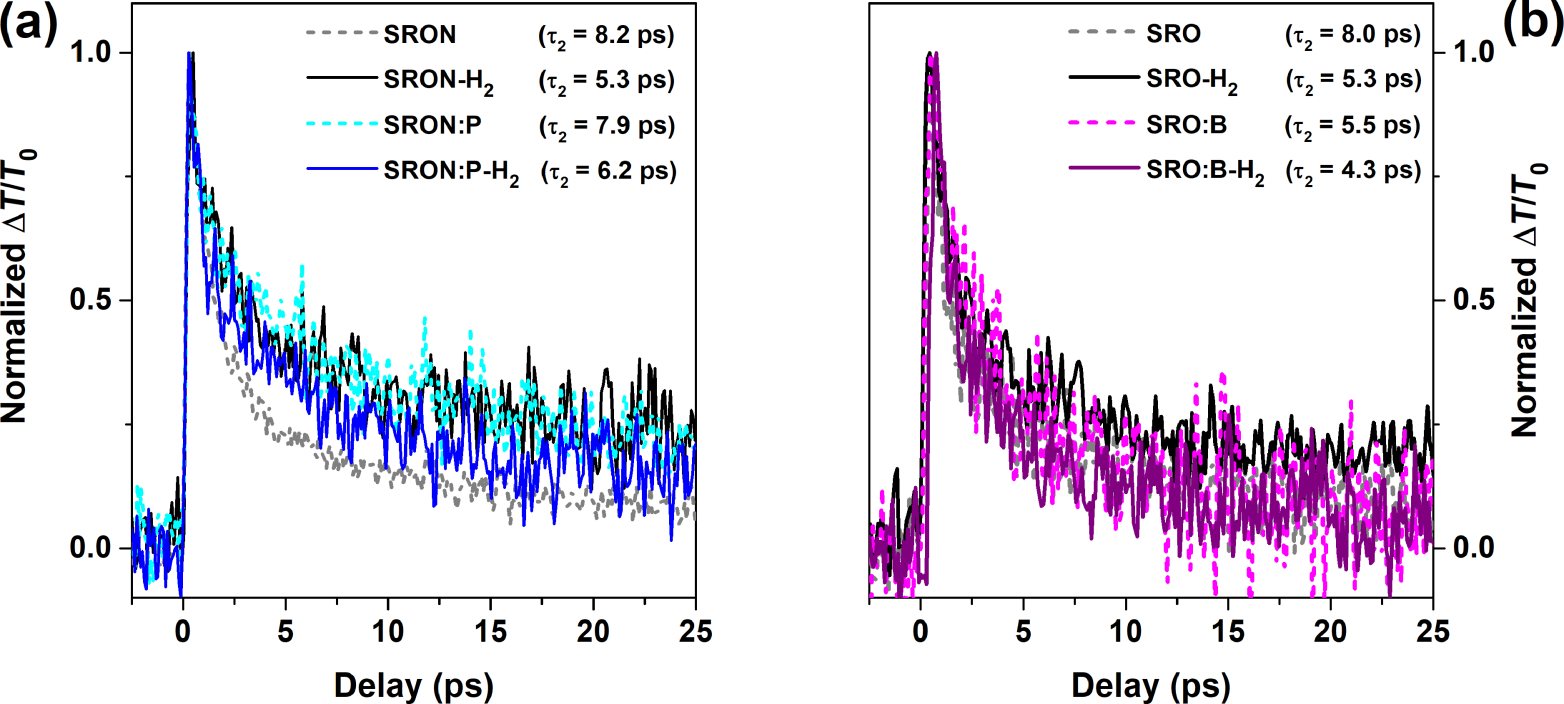
Transient transmission (TT) dynamics from pump-probe measurements at room temperature of ≈4.5 nm Si NCs from (a) SRON:P and P-free SRON and (b) SRO:B and respective B-free SRO. All samples are measured before and after H_2_-passivation. The curves are normalized for better comparability of the decay dynamics (although no significant or even doping-related differences exist in the initial signal intensity of the samples). The time constants of the component τ_2_ of the two-exponential fits (not shown for clarity) are given in the figure. The presence of P- or B-atoms in the NCs does not increase the speed of carrier recombination, as would be expected from the presence of dopant-induced free carriers.

In Figure 5 we report the transient transmission dynamics of samples with 4.5 nm Si NCs made of (a) SRON:P with 0.71 atom % P and (b) SRO:B with 1.32 atom % B, i.e., samples with substantial incorporation of dopant atoms and significant PL quenching. For this measurement the excitation pump pulse wavelength was 400 nm (efficiently absorbed by the NCs) and the probe pulse wavelength was 800 nm, which is hardly absorbed by the NCs. However, if free carriers are present in the NCs, whether from optical excitation or from doping, the probe light is absorbed. The transmission of the sample at the probe wavelength in the unexcited state is measured as *T*_0_ and the transmission as function of delay time between pump and probe (in steps of ≈100 fs) is plotted as


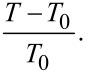


The pump flux is chosen to generate only a few excitons per NC [45]. Specifically, 2.3 mJ/cm^2^ (SRON) and 3.4 mJ/cm^2^ (SRO) were used, which correspond to the excitation regime with normal Auger recombination of excitons, excluding bimolecular recombination [46]. If an additional free carrier (electron from P-donor or hole from B-acceptor) would be present in a Si NC, the generated exciton(s) could efficiently and quickly recombine with the unpaired charge carrier via an Auger process. This would substantially accelerate the reduction of the total carrier density and a doped sample would become transparent in shorter time as compared to an intrinsic sample. It is obvious from Figure 5a and 5b that neither for SRON:P nor for SRO:B accelerated TT-dynamics exist. When fitting the curves, best results are obtained for a two-exponential fit


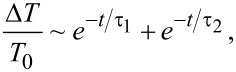


where the fast component τ_1_ ≈ 0.5 … 1.5 ps for all samples is attributed to ultrafast carrier trapping and thermalization events. The long component τ_2_ is associated with the actual Auger recombination of excitons and ranges from 5 to 8 ps without differences between doped or undoped samples. We conclude that no measurable initial carrier densities exist at room temperatures in P- or B-doped Si NCs in silicon oxide matrix.

The TT-results are presented for both H_2_-passivated and unpassivated states without distinctive differences, but one remark concerning the interaction of Si-doping and hydrogen shall be made: While P in the Si NC system is known to passivate dangling bonds (DBs) at the Si/SiO_2_ interface [7,42] similar to a post-annealing in H_2_, hydrogen treatments have also been shown to deactivate P-donors and B-acceptors in heavily doped Si nanowires [47] and in the bulk [48–50]. However, this type of dopant passivation solely relies on very reactive atomic hydrogen (rather than molecular H_2_ gas) and requires much lower temperatures of 100–150 °C to be efficient. When considering H_2_ as used in our work, the effective (endothermic) dissociation enthalpy of the reactions 2 P-DB + H_2_ → 2 P-H and 2 Si-DB + H_2_ → 2 Si-H yield ca. 0.05 eV and ca. 0.09 eV per DB passivation, respectively [51]. This finding renders the P–H bond breakage to occur at significantly lower temperatures as used at H_2_ anneals to passivate Si-DBs (450–500 °C).

Such a H-passivation mechanism of dopants requires their substitutional incorporation, which occurs apparently only in very small fractions for dopants in Si NCs (see section Electrical properties below). Therefore, neither from experimental evidence nor from fundamental considerations, it can be argued that the doping effect of P or B in Si NCs is obscured by H_2_-passivation. In contrast, the passivation of DB-defects at the Si/SiO_2_ interface often improves the interpretability of the measured data.

### Electrical properties

**Figure 6:**
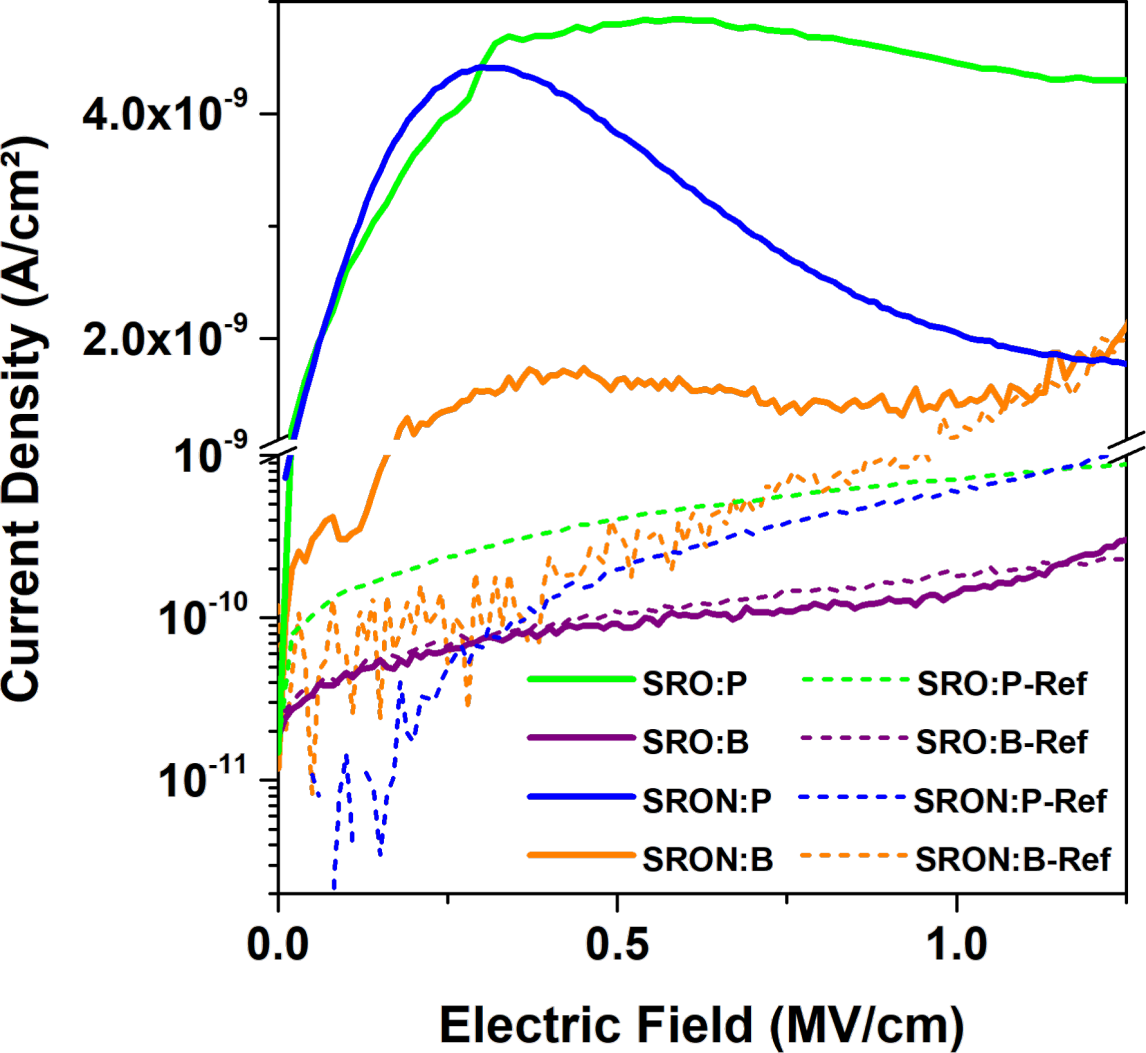
*J*–*E* data of injection-blocking MOS-capacitors with P- or B-incorporating Si NCs in SRO and SRON and their directly corresponding references (SRO:P with 0.59 atom %, SRON:P with 0.48 atom %, SRO:B with 1.32 atom %, SRON:B with 0.14 atom %).

If free charge carriers would be present in the Si NCs, or if they are generated via ionization by an external electrical field, it is possible to detect their presence by *I*–*V* measurements on MOS-capacitors with additional injection barriers [52–53]. Respective samples (injection-blocking MOS-capacitors) were fabricated with 10 nm-thick SiO_2_ buffer and capping layers to prevent low-field injection of carriers from either substrate or gate, so that only transient displacement currents are measured. The current density over electric field (*J*–*E*) curves of B- or P-incorporating SRO and SRON samples, together with dopant-free reference samples, are depicted in Figure 6. None of the reference samples (dashed lines) shows a current peak in the low E-field regime, which excludes significant contributions to the displacement current by undoped Si NCs or their host matrices (pure oxide vs oxynitride). The *J*-curves of the P-incorporating NCs show a broad peak at ≈0.5 MV/cm for SRO:P and a sharper peak at ≈0.3 MV/cm for SRON:P. The peak character for SRO:P is less clearly expressed. Whereas the rising shoulders of both *J*-peaks are quite similar, only for the SRON:P sample the current density decreases behind the peak with a comparable slope but remains on a plateau for SRO:P. The origin of the *J*-signal is the ionization of substitutional P-atoms in Si NCs and the subsequent accumulation of the “free” charge carriers under the gate blocking oxide (cf. [29] and [52] for details). Following the calculations therein, we can estimate the P-ionization energy of the *J*-peak (or respectively the beginning of the *J*-plateau) to ≈200 meV, in accordance with literature values on ionization energies of nano-sized Si [54–55]. For SRO:P the *J*-plateau indicates a broader distribution of P-ionization energies towards even larger values.

The comparison between SRO:B and SRON:B is even more surprising: While Si NCs from SRO:B do not show a *J*-peak but rather identical *I*–*V* behaviour as the respective reference, the SRON:B sample shows a weak plateau-like peak at ≈0.4 MV/cm. It is emphasized that the *J*-peak is found in the maximum possible B-doped SRON sample, but not in the max-doped SRO:B sample, despite an almost one order of magnitude higher B-concentration in the latter. Hence, B-doped Si NCs may be field-ionized in SRON, in contrast to our previous report on SRO [30] and in accordance with [53]. The hole-tunnelling properties of the matrix seem to determine the visibility of this effect and oxynitride seems to allow for a better hole transport (maybe assisted by N-related states), while N-free pure oxide apparently camouflages the field-ionization of B in SRO:B. In the case of electrons from field-ionized P-donors the situation is less critical since the conduction band offset of Si NCs and SiO_2_ is only about half that of the valence band offset [56].

**Figure 7:**
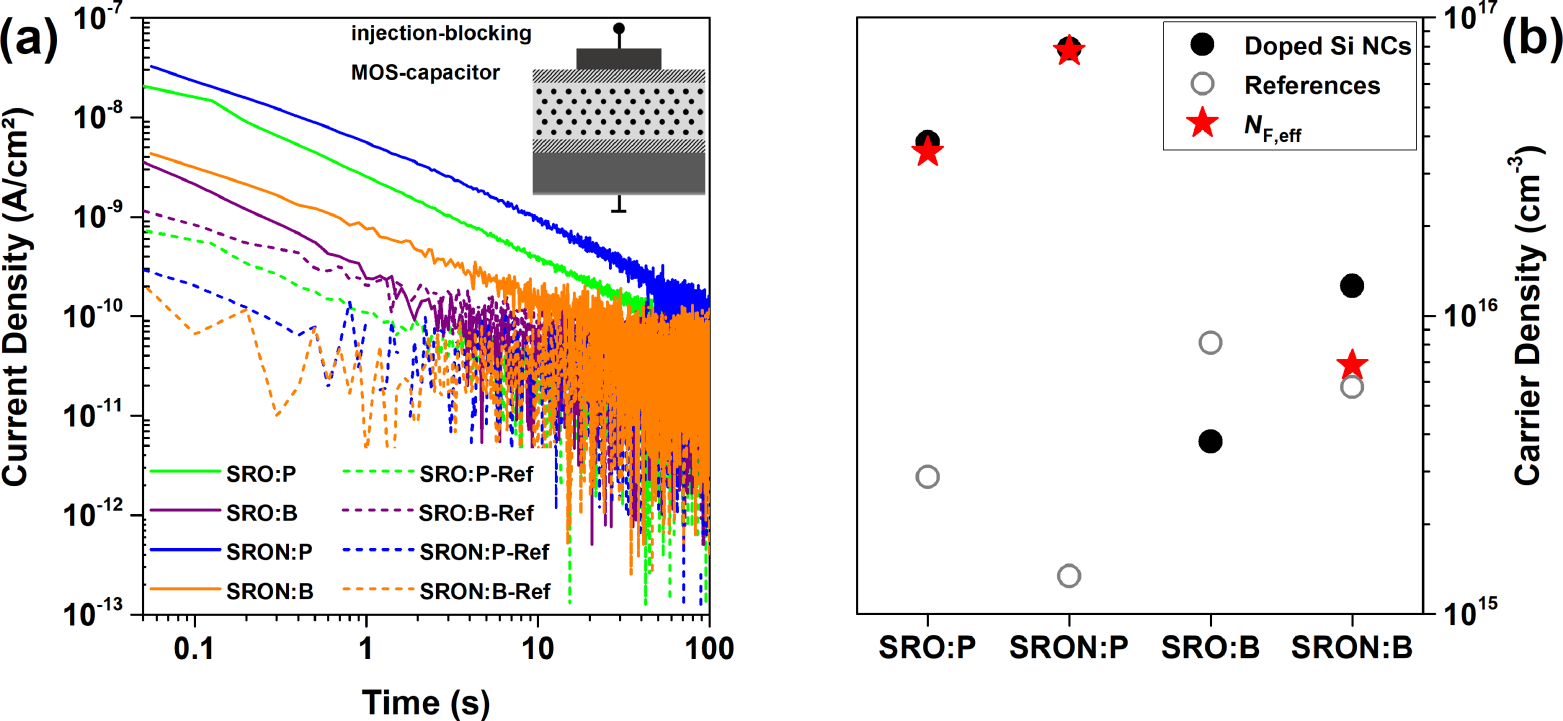
(a) *J*–*t* transients of the same MOS-capacitors as in the previous figure measured at *E* = 0.2 MV/cm. The inset shows a schematic cross-section of the device under test. (b) Free carrier density from field ionization of substitutional dopants derived from integrating transient current over time. For effective free carrier values (*N*_F,eff_) the values of the dopant-free references values are subtracted from the values of the doped Si NC samples.

Figure 7a shows *I*–*t*-transients of the injection-blocked MOS-capacitors (the inset depicts a schematic cross-section) measured at 0.2 MV/cm, i.e., at the onset of the *J*-peak/plateau (if present). As expected from the device geometry, all transient displacement currents reach the noise level at the minimum detectable limit (sub-pA range), which marks the end of the measurements. Whereas the fast drop of *J* of the reference samples within the first seconds of the measurement is attributed to dielectric relaxation, the P-doped Si NC samples clearly show mobile charge redistributions on a longer timescale. For the B-doped Si NCs the situation is less clear, since the noise level is reached earlier. A likely cause for this behaviour might be a lower density of redistributable charge. With the exception of SRO:B samples, there is also a 1–2 orders of magnitude higher *J*-level throughout a major part of the transient period between the doped samples and their respective references. By integrating the measured current over time, the corresponding total charge, generated by field ionization of dopants on Si-lattice sites in the NCs, can be estimated [52]. The free carrier densities of all samples at 0.2 MV/cm are shown in Figure 7b. Values of (4 ± 3) × 10^15^ cm^−3^ were obtained for the reference samples (grey open circles); we note that these values are strongly influenced by dielectric relaxation. From the doped samples (black filled circles) only SRO:B has a similar value (being slightly below its reference). All other doped NC-samples have free carrier concentrations in the 10^16^ cm^−3^ range. In order to exclude a contribution to the free carrier values from the dielectric relaxation, we subtract the reference-values to obtain the effective free carrier density (*N*_F,eff_, red spheres in Figure 7b). It is obvious that P dominates over B and SRON over SRO: Sample SRON:P has about twice the integral charge than SRO:P and SRON:B is an order of magnitude lower than SRON:P. In this context, point out that SRON:B has a B-concentration that is just 30% of the P-concentration in SRON:P. Although the initial dopant concentration in the Si-rich oxide is not the figure of merit but the substitutional incorporation in the NCs, these results still indicate that B-doping is less efficient than P-doping. This is underlined by the electrical properties of the SRO:B samples, which do not even have a positive effective free carrier density. Here, the very small effective free carrier density of SRO:B is exceeded by the carrier density of SRO:B-Ref, which might originate from the slightly different NC-density caused by the B-dependent Si-content (cf. Figure S1, Supporting Information File 1). We note that for SRON:P a field ionization doping efficiency of ≈4% was derived by dividing *N*_F,eff_ with the number of P-atoms in the NCs measured by APT [29], which allows to estimate for Si NCs from SRO:P a field ionization doping efficiency of ≈2%, whereas for SRON:B in absence of measurable APT results no efficiency can be estimated.

## Conclusion

Comparing oxynitride and N-free oxide as matrix for P- or B-incorporating Si NCs, no significant differences were observed structurally (SIMS, APT) or optically (PL, TT). Electrically (*I*–*V*, *I*–*t*) differences occur, which appear to be related to the insulating nature of the oxide matrix itself and the respective band offsets. For both dopants a slight room-temperature PL quenching is observed, becoming strong only for dopant concentrations beyond 1 atom %. This circumstance together with the absence of the spectral behaviour expected for doped NCs indicates that dopant-induced defects are the origin of PL quenching, in accordance with theoretical DFT predictions. Low-temperature PL spectroscopy and transient transmission measurements show no indications for dopant-induced free carriers in Si NCs. Electrical measurements on MOS-capacitors with additional injection blocking layers prove that E-fields in the range of 0.3–0.5 MV/cm are required to ionize the small fraction of lattice-incorporated dopants and to generate charge carriers. It was shown that the higher resistivity of the N-free oxide as compared to oxynitride masks the field-induce charge carrier generation from B-doped NCs. Comparing P-doped NCs in both matrices this effect was not found.

Summarizing the results reported here and previously [29–30,41,52] it turns out that P- and B-dopants in oxide-embedded Si NCs remain predominantly on interstitial lattice sites where they cannot be ionized by thermal energy at room-temperature, in agreement with the nanoscale-effects of self-purification, quantum- and dielectric confinement. This results in diminutive doping efficiencies [57]. We note that broader NC size distributions with tails towards the ≈10 nm range [58] or percolated nano-Si networks [53] are not subject to the same strong confinement conditions, so that measurable free carrier densities are likely.

The fundamental inability of efficient conventional impurity doping at the bottom end of the nanoscale requires different doping approaches that either relocate the dopants in the surrounding matrix (e.g., Si modulation doping by SiO_2_:Al) [59] or do not require impurities at all (e.g., electrically reconfigurable nanowire-FETs [60] or *p*/*n*-behaviour induced by energy offsets created by locally Si_3_N_4_ and SiO_2_ embedded Si-nanowires [61]).

## Supporting Information

File 1 Additional figures.
