# Supplementary material for: Absence of free carriers in silicon nanocrystals grown from phosphorus- and boron-doped silicon-rich oxide and oxynitride
Source: Beilstein J Nanotechnol. 2018 May 18;9:1501–11. doi: 10.3762/bjnano.9.141 (PMC6009393; doi:10.3762/bjnano.9.141)
Supplement: File 1 — Additional figures. [file Beilstein_J_Nanotechnol-09-1501-s001.pdf]

Supporting Information

for

Absence of free carriers in silicon nanocrystals grown from phosphorus- and boron-doped silicon-rich oxide and oxynitride

Daniel Hiller*[1,2], Julian López-Vidrier[2], Keita Nomoto[3], Michael Wahl[4], Wolfgang Bock[4], Tomáš Chlouba[5], František Trojánek[5], Sebastian Gutsch[2], Margit Zacharias[2], Dirk König[6], Petr Malý[5], and Michael Kopnarski[4]

Address: [1]Research School of Engineering, Australian National University (ANU), Canberra, Australia, [2]Laboratory for Nanotechnology, Department of Microsystems Engineering (IMTEK), University of Freiburg, Germany, [3]The University of Sydney, Faculty of Engineering and Information Technologies, School of Aerospace, Mechanical and Mechatronic Engineering, Sydney, Australia, [4]Institute for Surface and Thin Film Analysis GmbH (IFOS), Kaiserslautern, Germany, [5]Department of Chemical Physics and Optics, Charles University, Prague, Czech Republic and [6]Integrated Materials Design Centre (IMDC), University of New South Wales (UNSW), Sydney, Australia

* Corresponding author: daniel.hiller@anu.edu.au

Additional Figures

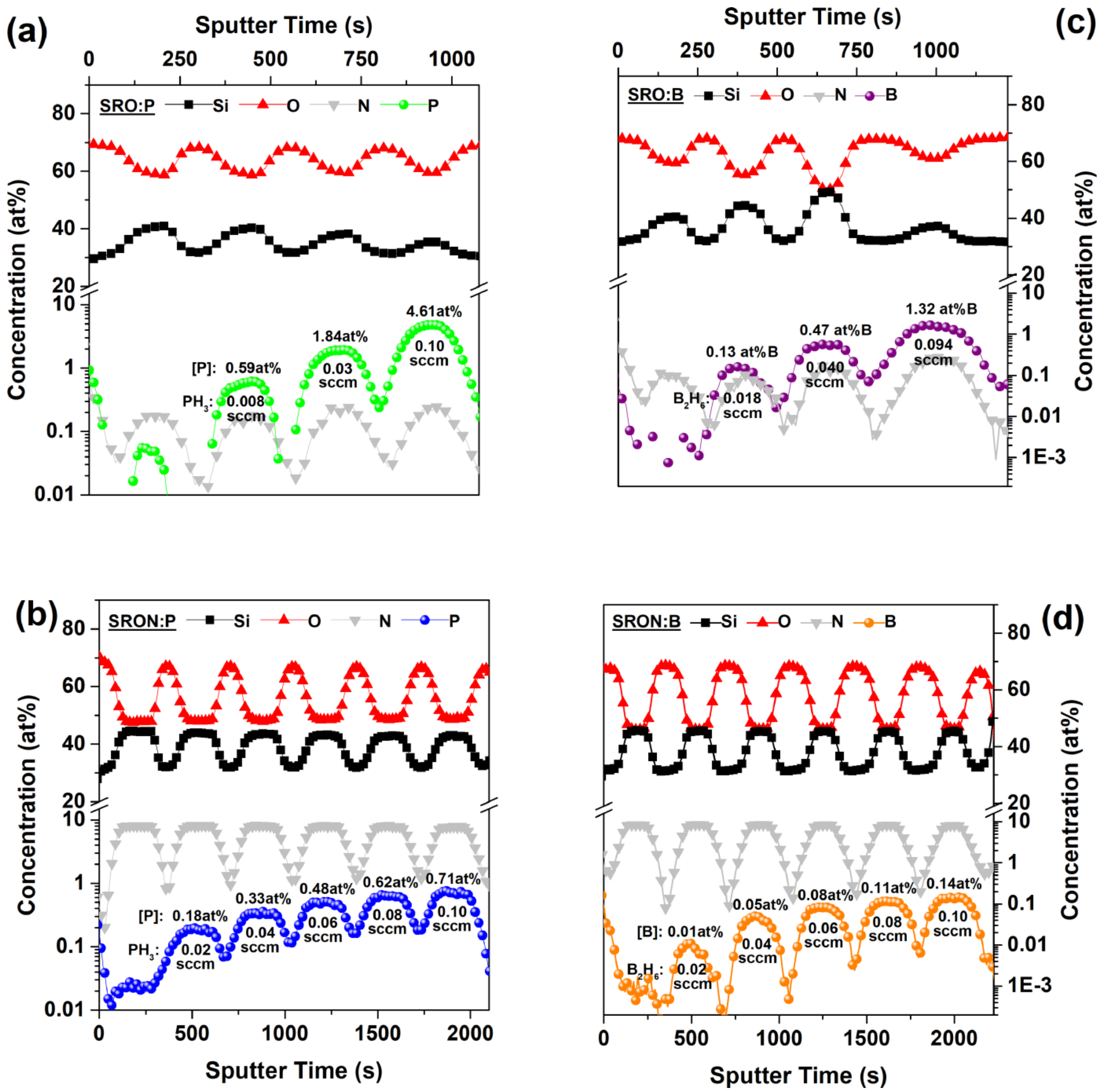

Figure S1: Quantitative composition as measured by MCs$^+$-SIMS of: (a) SRO:P and (b) SRON:P for different PH_3 fluxes, and (c) SRO:B and (d) SRON:B for different B_2H_6 fluxes. All samples are not annealed and the data is corrected for the ~5 at% (SRO) or ~10 at% (SRON) of H, which would readily effuse during the annealing to form Si NCs. The topmost layers are the respective intrinsic material, deposited without any dopant gas flow. The measured background P-concentration of <0.05 at% is attributed to potential mass interferences of ^{31}P and ^{30}SiH, while the B-background concentration around 10^{-3} at% represents the detection limit.

In the P-doped Si-rich layers the Si-concentration (black squares) decreases just slightly with increasing P-concentration for SRON:P (~44 to ~43 at%) and more significantly for SRO:P (~41 to ~35 at%). However, the *missing* Si equals the concentration of additional P in the layers, i.e., during deposition of P-doped Si-rich oxides P replaces Si, which will decrease the size and areal density of the Si NCs to be formed from such layers. In contrast, the O-concentrations (red triangles) of SRO:P, or respectively, the (O+N)-concentration of SRON:P remain almost constant. The same observations can be made for the B-doped Si-rich oxides, though the influence of B_2H_6 on the Si- and O-concentration in SRO:B appears to be more severe compared to SRO:P, while for SRON:B virtually no Si- and O-concentration change by B_2H_6 is observed.

We use the measured Si- and O-concentrations of SRO:B to approximate corrected PL-intensities in Fig. 3(c). This is done by assuming complete phase separating into Si and SiO_2 during annealing. The excess-Si content not bound to oxygen is normalized to the reference SRO-sample without boron. The resulting factor is multiplied with the PL-intensity to account for the missing/additional excess-Si present in the SRO that forms the luminescing NCs during annealing.

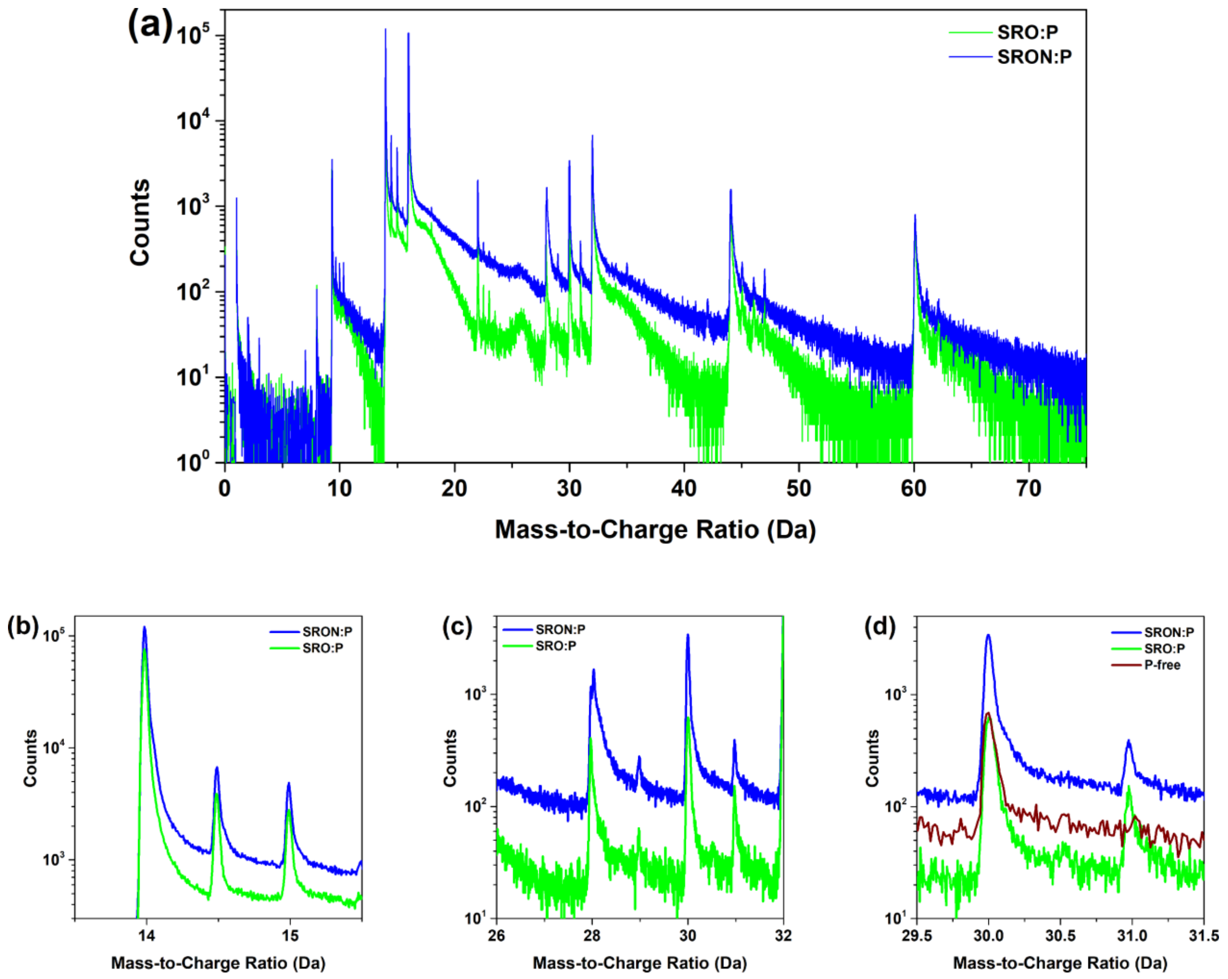

Figure S2: Mass spectra of the APT measurements: (a) Broad range spectrum up to 75 Da. (b) The regions around 14 Da (attributed to $^{14}N^{+}$ or $^{28}Si^{2+}$) and (c) around 28 Da (attributed to $^{14}N_2^{+}$ or $^{28}Si^{+}$), which indicate only a very small influence of N on the mass spectra. (d) The deceptive attribution of $^{31}P^{+}$ by the detection of significant amounts of $^{30}Si^{1}H^{+}$ or $^{30}Si^{16}O_2^{2+}$ is ruled out by a P-free reference sample that shows no signal at 31 Da.

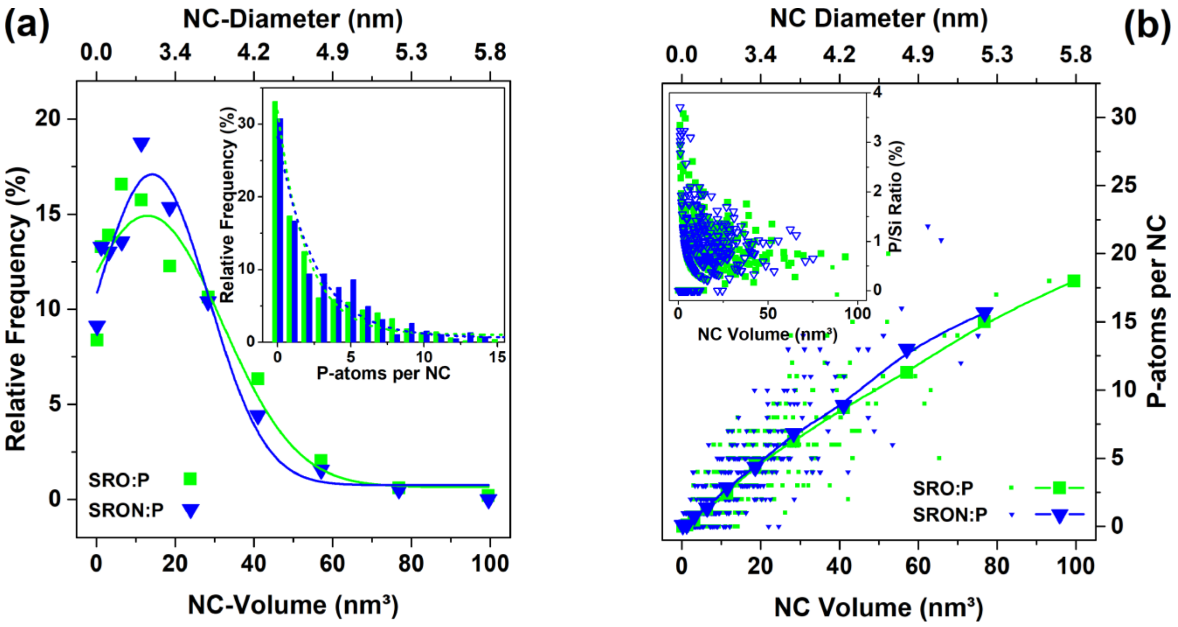

Figure S3: Analyses based on APT data: (a) Size distribution of the Si NCs in pure oxide with 0.59 at% P and oxynitride matrix with 0.71 at% P (solid lines are Gaussian fits). Since both size distributions can be well fitted with Gauss functions with equal mean NC-diameters of ~3 nm, the slightly different excess-Si concentrations in SRO:P and SRON:P as well as the nitrogen, have obviously no influence on the mean NC-size and the size distribution. The inset depicts the frequency of NCs that incorporate *n* P-atoms. The dashed lines are exponential probability fits, $F_{rel}(n) = \lambda \cdot e^{\lambda n}$, where λ denotes the inverse of the expected value.
(b) The number of P-atoms incorporated in one NC as function of NC-size (small data points: APT raw data; large data points: averaged values derived by grouping into volume classes; solid lines (splines) are just a guide to the eye). In inset of (b) atomic ratio of P over Si from the APT cluster analysis is plotted over the NC-volume. The average P-concentration is 0.64% for SRO:P and 0.79% for SRON:P, i.e., well in accordance with the MCs^+-SIMS values.

The lower x-axes of both graphs are the NC-volumes computed via the iso-concentration surfaces, while for the upper x-axes (in good approximation to reality) spherical NC-shapes are assumed. The first data point corresponds to 0.22 nm³ (0.75 nm diameter), which is below the typical crystallization limit and might indicate amorphous nanoclusters. Alternatively, the non-unity atom detection efficiency of APT and resolution limiting APT-artefacts make these structures appear smaller than they are.

According to APT every NC incorporates on average 2-3 P-atoms though almost $^1/_3$ of the NCs are P-free. Whereas Fig. S3(b) proves that larger NCs contain more P-atoms than small NCs, the inset demonstrates that the P/Si-ratio is highest for the small NCs. In any case, the distribution of P-atoms over the NCs is strongly non-homogenous: Si NCs of ≥4 nm in diameter incorporate at least 5 P-atoms or more, while NCs incorporating just 1 P-atom are on average 2.2–2.3 nm in diameter. The largest P-free NCs that were measured are ~3 nm in diameter but their mean size is only 1.6–1.7 nm. The P-concentration of the subset of P-incorporating NCs of both samples is on average ~3×10^{20} cm^{-3} which is very close to the solubility limit of P in Si for the annealing temperatures used here (1100°C for SRO, 1150°C for SRON) [1]. Hence, most Si NCs incorporate P up to the solubility limit, while less than 20% exceed the solubility limit according to APT.

We are aware that the detection of single P-atoms is subject to uncertainties of the APT measurement such as the noise level of the analysis, the effect of thermal tails in the mass spectra and LME artefacts, but the direct comparison of SRO:P and SRON:P samples is not affected by that and the agreement of the total P-concentration with SIMS data supports the assumption of a reliable APT-data treatment.

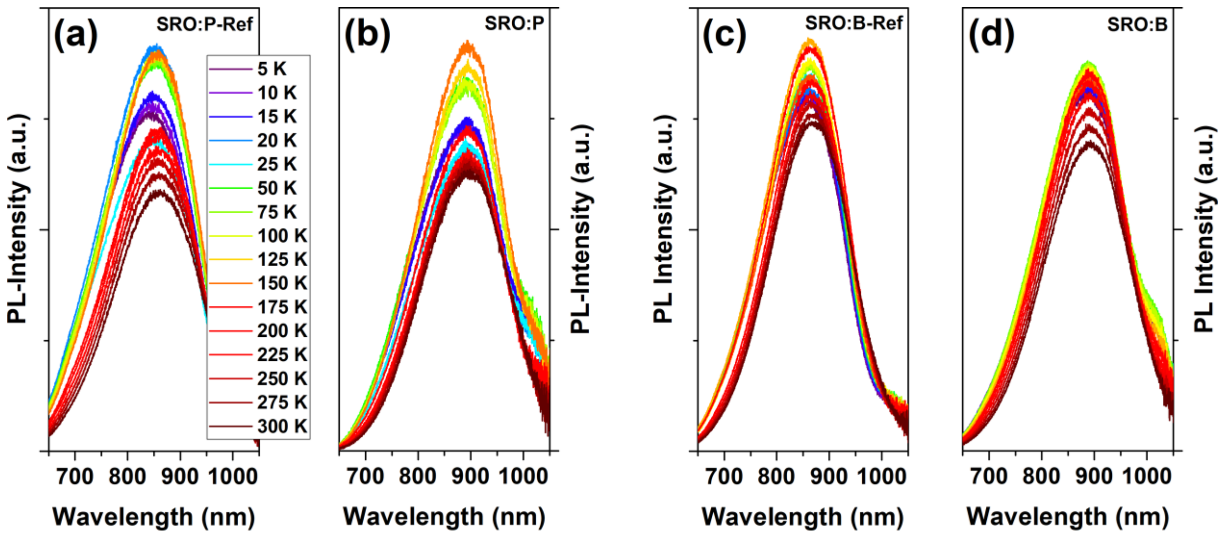

Figure S4: Temperature dependent PL spectroscopy from 5 to 300 K: The samples consist of superlattices with 5 nm SRO (a) undoped reference and (b) 0.59 at% P, (c) undoped reference and (d) 0.47 at% B.

References

1. Borisenko, V. E.; Yudin, S. G. *Phys. Status Solidi A* **1987**, *101*, 123-127.
